# Development and validation of radiomics nomogram for metastatic status of epithelial ovarian cancer

**DOI:** 10.1038/s41598-024-63369-1

**Published:** 2024-05-30

**Authors:** Yinping Leng, Xiwen Wang, Tian Zheng, Fei Peng, Liangxia Xiong, Yu Wang, Lianggeng Gong

**Affiliations:** 1 Department of Radiology, The Second Affiliated Hospital of Nanchang University, Minde Road No. 1, Nanchang, 330006 Jiangxi China; 2 Clinical and Technical Support, Philips Healthcare, Shanghai, 200072 Shanghai China

**Keywords:** Computed tomography, Metastasis, Epithelial ovarian cancer, Radiomics, Nomogram, Cancer, Diseases, Oncology

## Abstract

To develop and validate an enhanced CT-based radiomics nomogram for evaluating preoperative metastasis risk of epithelial ovarian cancer (EOC). One hundred and nine patients with histologically confirmed EOC were retrospectively enrolled. The volume of interest (VOI) was delineated in preoperative enhanced CT images, and 851 radiomics features were extracted. The radiomics features were selected by the least absolute shrinkage and selection operator (LASSO), and the rad-score was calculated using the formula of the radiomics label. A clinical model, radiomics model, and combined model were constructed using the logistic regression classification algorithm. Receiver operating characteristic (ROC) curve analysis and decision curve analysis (DCA) were used to evaluate the diagnostic performance of the models. Seventy-five patients (68.8%) were histologically confirmed to have metastasis. Eleven optimal radiomics features were retained by the LASSO algorithm to develop the radiomic model. The combined model for evaluating metastasis of EOC achieved area under the curve (AUC) values of 0.929 (95% CI 0.8593–0.9996) in the training cohort and 0.909 (95% CI 0.7921–1.0000) in the test cohort. To facilitate clinical use, a radiomic nomogram was built by combining the clinical characteristics with rad-score. The DCA indicated that the nomogram had the most significant net benefit when the threshold probability exceeded 15%, surpassing the benefits of both the treat-all and treat-none strategies. Compared with clinical model and radiomics model, the radiomics nomogram has the best diagnostic performance in evaluating EOC metastasis. The nomogram is a useful and convenient tool for clinical doctors to develop personalized treatment plans for EOC patients.

## Introduction

Among gynecological tumors, ovarian cancer has the highest mortality rate, with epithelial ovarian cancer (EOC) being the principal cause of death^1,2^. Due to the anatomical location of the ovaries, early detection of EOC is challenging, resulting in approximately 70% of patients being diagnosed at advanced stages characterized by intra-peritoneal spread and distant metastases^3^. The five-year survival rate for patients in advanced-stage is less than 40%^4^. Early detection of metastases is important for clinicians to improve the accuracy of preoperative staging and guide individualized treatment plans for EOC^5^. For example, patients with low-risk early-stage ovarian cancer are expected to retain their fertility, whereas late-stage patients require surgical resection and adjuvant chemotherapy^6,7^. However, the gold standard methods for EOC staging are surgery and histopathologic diagnosis, so it is necessary to explore non-invasive techniques for preoperative metastasis risk assessment to assist clinical decision-making^8^.

Imaging methods such as positron emission tomography with CT (PET-CT), magnetic resonance imaging (MRI) and computed tomography (CT) have been used to assess EOC metastasis at the local, regional and distant levels. The diagnostic effectiveness of PET-CT in EOC is comparatively inferior to that of MRI and CT, exhibiting lower sensitivity and specificity^9^. MRI is characterized by a restricted scanning range, elevated cost, extended examination duration, and susceptibility to displacement. Consequently, in cases involving patients with peritoneal metastasis and prominent ascites, the diagnostic precision of MRI staging may be inferior to that of CT^10^. The European Society of Urogenital Radiology recommends CT as the preferred imaging method for preoperative staging and follow-up of EOC^11^. CT scanning is a rapid and widely used imaging technique that enables the assessment of the primary site and extent of tumor invasion, thereby providing assistance in formulating surgical treatment plans^9^. However, traditional CT evaluation continues to rely on radiologists’ professional knowledge and subjective experience to provide diagnostic information. Thus, it is critical to investigate a more objective and accurate method of assessing ovarian cancer metastasis in order to better guide the treatment plan and prognosis assessment.

Radiomics utilizes various algorithms to analyze image data from a specific region of interest, extracting radiomics features that include first-order, second-order, or higher-order data. This process aims to improve the accuracy of clinical diagnosis and prognostic value by uncovering deep level relationships within data^12–14^. By leveraging radiomics, it becomes possible to detect subtle structures and reveal hidden image information that may not be visible to the naked eyes, thereby strengthening the diagnostic and prognostic utility^15–18^. Encouragingly, CT-based radiomics has demonstrated success in the diagnosis, staging, assessment of lymph node metastasis, prediction of disease-free survival, evaluation of recurrence risk, and assessment of treatment response for various types of tumors^19–22^. Unfortunately, there is a paucity of studies focusing on personalized assessment models for preoperative metastasis risk in ovarian cancer.

Our study aimed to estimate the value of enhanced CT-based radiomics models in diagnosing preoperative metastasis in EOC patients. We aslo aimed to establish a radiomics nomogram to facilitate in treatment planning and decision-making.

## Materials and methods

### Patients

**Figure 1:**
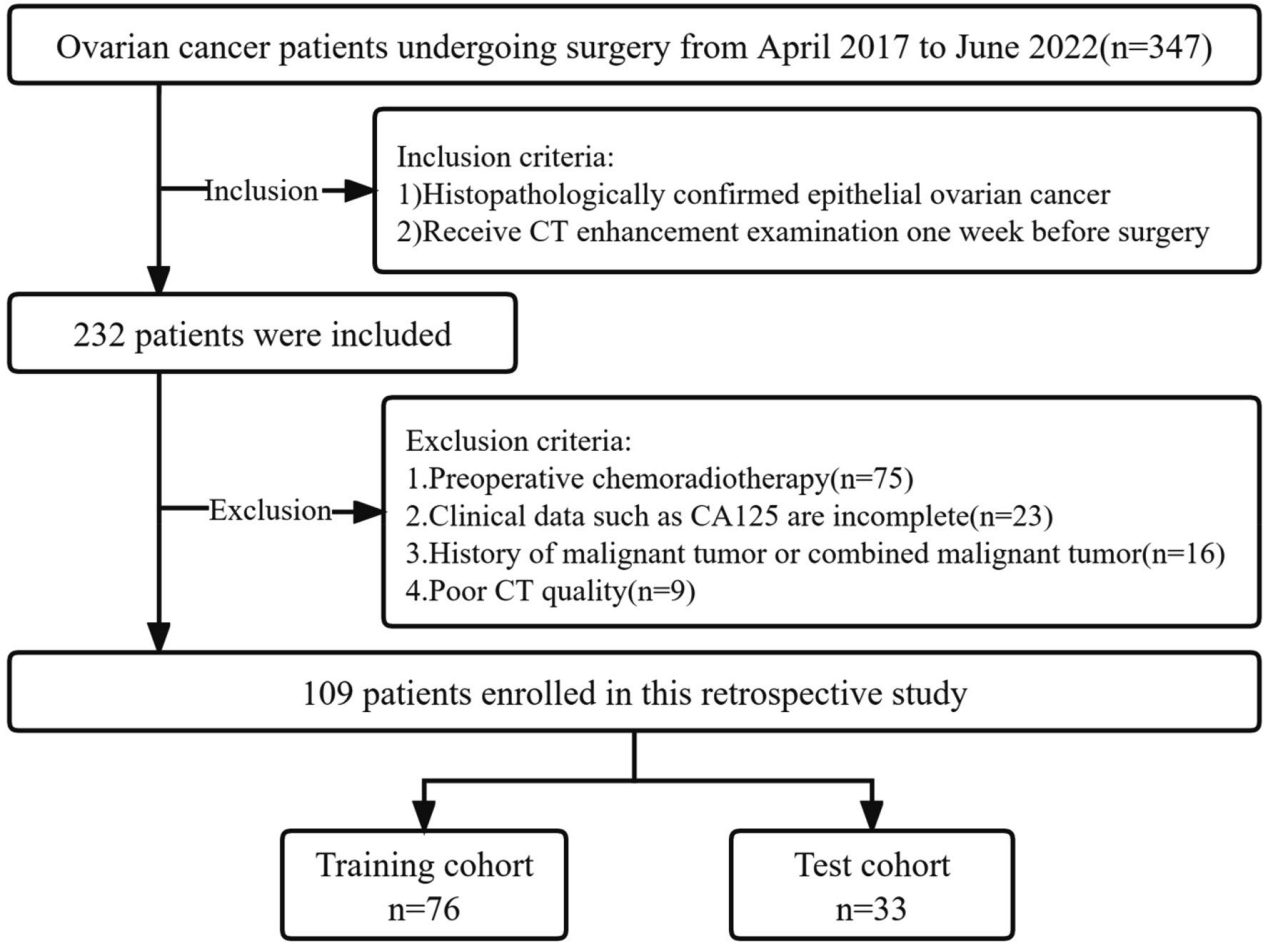
Flowchart of patient selection.

This retrospective study was reviewed and approved by the Institutional Review Board of the Second Affiliated Hospital of Nanchang University. Written informed consent for participants was not required for this study in accordance with the national legislation and the institutional requirements. All methods were performed in accordance with the relevant guidelines and regulations. The study population consisted of 347 patients diagnosed with EOC between April 2017 and June 2022. Patient data were obtained from our hospital's Picture Archiving and Communication System (PACS). We included 232 EOC patients who met the inclusion criteria, which required histological confirmation of EOC and the completion of CT enhancement examination one week prior to surgery. Among the 232 EOC patients, we excluded 123 individuals based on the following exclusion criteria: patients who received preoperative chemoradiotherapy (n = 75), those with incomplete clinical data such as carcinoma antigen 125 (CA125) levels (n = 23), patients with other malignant tumors or combined malignancies (n = 16), and those with poor CT image quality (n = 9). Finally, a total of 109 EOC patients were included in the final analysis. The flowchart of patient selection was shown in Fig. 1.

The clinical characteristics, including preoperative CA125 and carbohydrate antigen 199 (CA199) levels, age, International Federation of Gynecology and Obstetrics (FIGO) stage, histological type, tumor location, and menopausal status, were obtained from the medical records of the patients. The FIGO stage of EOC was determined according to the FIGO 2014 staging system^23^. The histopathological results of EOC and the metastasis status were gathered and utilized as the ground truths for patients in the development and validation of various models. The patients were randomly divided into training and test cohorts in a ratio of approximately 7:3.

### CT images acquisition and preprocessing

CT imaging was obtained using two different CT scanners: the Brilliance 16 (Philips Medical Systems, the Netherlands) and the SOMATOM definition flash (Siemens Healthcare, Munich, Germany). The scanning parameters of the Philips’ CT scanner were as follows: rotation time of 0.4 s; slice collimation of 64 × 0.625 mm; matrix size of 512 × 512; tube current of 250 mAs; pitch of 0.891; tube voltage of 120 kVp; slice thickness of 5 mm; and slice interval of 5 mm. The scanning parameters of the Siemens’ CT scanner were as follows: rotation time of 0.5 s; slice collimation of 128 × 0.6 mm; matrix size of 512 × 512; tube current of 200 mAs; pitch of 0.6; tube voltage of 120 kVp; slice thickness of 2 mm; and slice interval of 1 mm. After a plain scan, an iodinated contrast media (370 mg I/mL) was administered via the left anterior elbow vein using a high-pressure syringe (Ulrich, German) at a flow rate of 4.0 mL/s. The enhanced arterial phase and venous phase images were acquired with a delay of 25-30 s and 60-70 s, respectively.

The CT images were exported in DICOM format, and folders were created with uniform names. Before image segmentation, preprocessing was required. All CT images were preprocessed in Python 3.9. The images were resampled to 1.0 × 1.0 × 1.0 mm^3^ through linear interpolation. In the preprocessing stage, image resampling is conducted to ensure that annotation results are consistent across the different image level and that the smoothness of masks remains uniform.

### Tumor segmentation and feature extraction

Two observers (L.Y.P and W.X.W, with 3 and 8 years of gynecologic diagnosis experience, respectively), independently analyzed the preprocessed CT images of all patients. Both observers were blinded to the histological results. In cases where there was disagreement between the two observers, a third experienced radiologist (G.L.G) with 35 years of gynecological diagnosis experience was consulted to provide a final opinion. After performing CT evaluations to identify the tumor area, one of them (L.Y.P) was arranged to segment the tumors. The volume of interest (VOI) was manually delineated layer by layer using the 3D slicer software (version 4.13.0; www.slicer.org). During the segmentation process, care was taken to avoid including blood vessels and other abdominal organs.

Discretize voxel intensity values using a fixed bin width of 25 HU. This can reduce computational complexity and help mitigate the impact of noise on feature extraction. Normalize the signal strength of the image to 1–500 HU. A total of 851 radiomics features were extracted automatically from the VOI using the pyradiomics plug-in integrated within the 3D slicer software (version 3.0.1). The radiomics features included first-order features (n = 18); shape features (n = 14); textural features (n = 75) extracted from the higher-order statistical methods, such as gray-level run length matrix (GLRLM) features, gray-level dependency matrix (GLDM) features, gray-level co-occurrence matrix (GLCM) features, neighborhood gray-tone difference matrix(NGTDM) features, gray-level size zone matrix(GLSZM) features; wavelet features (n = 744), which were generated from the eight derived images using Haar wavelet decomposition of first-order statistics and texture features.

To evaluate the reproducibility and accuracy of the extracted feature values, a subset of 20 patients was randomly selected. This patients underwent tumor segmentation by both radiologists one month later. Subsequently, intra-observer consistency analysis was conducted by comparing the VOIs drawn by the same observer, and inter-observer consistency analysis was performed by comparing the VOIs drawn by the two observers.

### Feature selection and model construction

The intra-class correlation coefficient (ICC) was conducted to assess the consistency between the two observers in analyzing radiomics features. Only the features with good consistency (ICC > 0.75) were selected for further analysis. The radiomics features of the training cohort were standardized through Z-score normalization. To reduce the dimensionality of radiomics features, we employed a two-step approach to select the features within the training cohort. Firstly, a multivariable ranking algorithm called minimum redundancy maximum relevance (mRMR) was employed based on the heuristic scoring criteria, to recognize the most important features. Only the top-ranked features with maximum correlation and minimum redundancy were retained. Subsequently, the least absolute shrinkage and selection operator (LASSO) regression method is used to select features. The grid search method is used to find the optimal regularization parameter lambda (λ). Nonzero coefficient features were selected and their linear combination was calculated to generate radiomics signatures. Radiomics score (rad score) was acquired for each patient.

**Figure 2:**
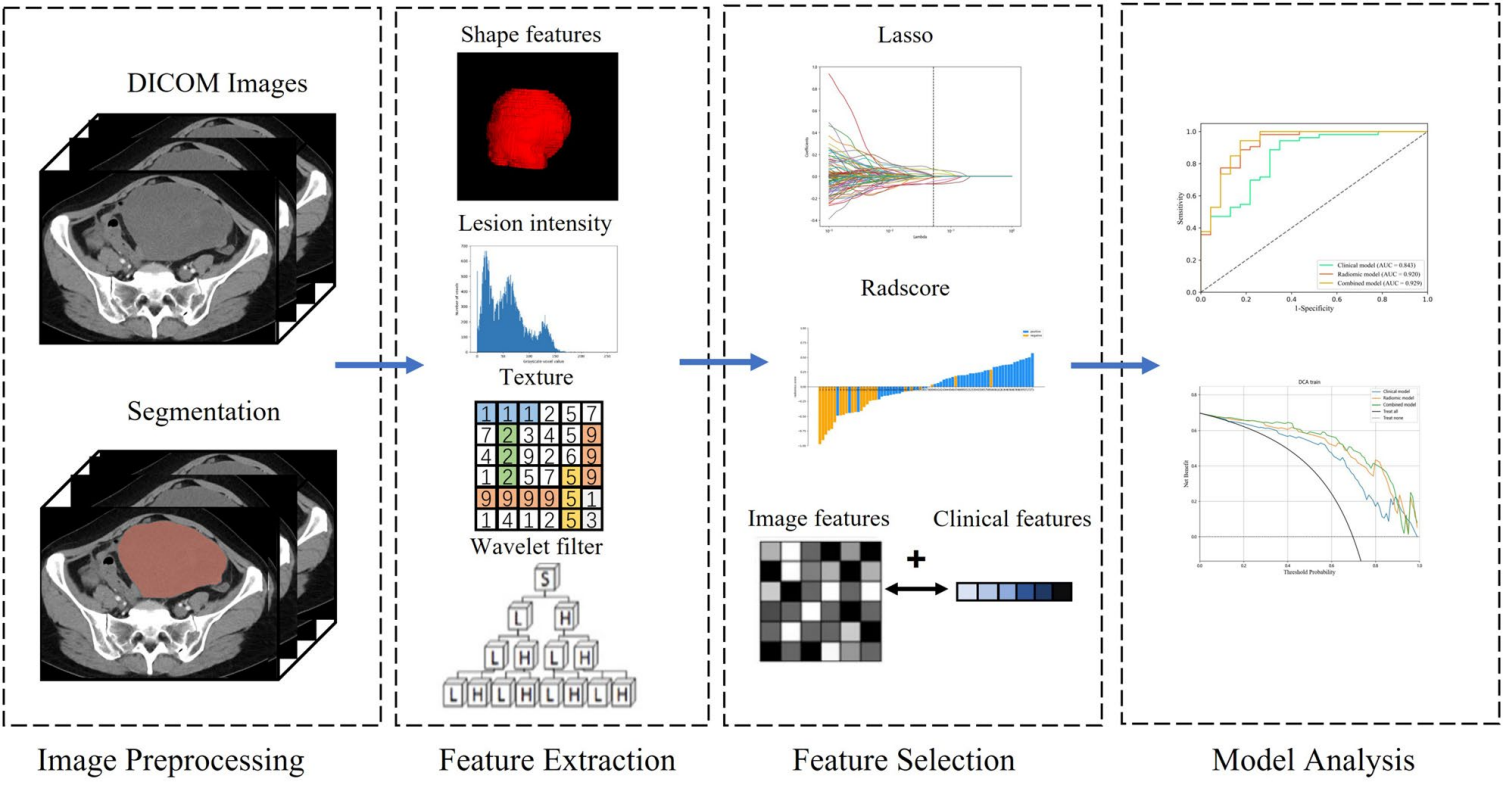
Radiomics workflow in our study.

Identify the most relevant clinical characteristics associated with EOC metastasis through univariate logistic regression and LASSO methods, and establish a clinical model. The rad score was combined with above clinical characteristics to develop a combined model. Finally, we construct a visual radiomics nomogram based on the combined model. This nomogram provides a graphical representation of the predictive model, incorporating both radiomics and clinical factors. The workflow is shown in Fig. 2.

### Model evaluation

The evaluation indicators of the models include receiver operating characteristic (ROC) curves, area under the curve (AUC), sensitivity, specificity, and accuracy. To evaluate the clinical utility of the radiomics nomogram, the net benefits of each model at different threshold probabilities was calculated by decision curve analysis (DCA). The goodness of fit of the nomogram was assessed via the Hosmer–Lemeshow test. Additionally, calibration curves were ultilized as a visual tool to assess the agreement between predictions and observations of the predicted values.

### Statistical analysis

SPSS 26.0 (IBM), Python (version 3.9) and R software (version 4.2.1) were used for statistical analysis. Qualitative data were compared using the chi-square test or Fisher’s exact test. Quantitative data were tested for normal distribution using Kolmogorov–Smirnov test. Quantitative data conforming to the normal distribution were expressed as mean ± SD, and the t-test was used for comparison between groups. Quantitative data of non-normal distribution were expressed as medians with interquartile ranges, and the Mann–Whitney U-test was used for comparison. A *P*-value < 0.05 was considered significant. The package versions of Python and R were presented in the Appendix material, Appendix A1.

### Ethics approval

The studies involving human participants were reviewed and approved by the Institutional Review Board of the Second Affiliated Hospital of Nanchang University. Since data were evaluated retrospectively, pseudonymously and were solely obtained for treatment purposes, a requirement of informed consent was waived by the Institutional Review Board of the Second Affiliated Hospital of Nanchang University. All methods were performed in accordance with the relevant guidelines and regulations.

## Results

### Clinical characteristics

The clinical characteristics were listed in Table 1. The average age was 55.96 ± 9.84 years. No significant differences were observed between the two groups in terms of age, CA199 and menopause state. There were significant differences between the two groups in terms of FIGO stage, histological type, CA125, and tumor location. The characteristics of all patients in the training and test cohort were shown in Table 2. There were no significant differences between the training cohort and the test cohort in clinical characteristics.

**Table 1:** The clinical characteristics of all patients.

Characteristics	Metastatic (n = 75)	Non-metastatic (n = 34)	*P-*value
Age (mean ± SD)	56.67 ± 10.08	54.41 ± 9.25	0.270^1^
FIGO stage (%)			< 0.001^2^
I	0	27 (79.4%)	
II	4 (5.3%)	7 (20.6%)	
III	62 (82.7%)	0	
IV	9 (12%)	0	
Histological type (%)			< 0.001^2^
HGSC	70 (93.3%)	16 (47.1%)	
Non-HGSC	5 (6.7%)	18 (52.9%)	
Menopause (%)			0.868^2^
Yes	54 (72%)	25 (73.5%)	
No	21 (28%)	9 (26.5%)	
CA125 [median (IQR),U/ml]	1164.7 (259.5–3673.0)	186.1 (116.0–415.2)	< 0.001^3^
CA199 [median (IQR),U/ml]	14.6 (8.8–27.6)	15.0 (7.0–76.7)	0.478^3^
Location (%)			< 0.001^2^
Unilateral	29 (38.7%)	27 (79.4%)	
Bilateral	46 (61.3%)	7 (20.6%)	

*SD* standard deviation, *FIGO* International Federation of Gynecology and Obstetrics, *HGSC* high-grade serous carcinoma, *CA199* Carbohydrate antigen 199, *CA125* carcinoma antigen 125, *IQR* interquartile range.

^1^Student t test.

^2^Pearson’s chi-square test or Fisher test.

^3^Mann-Whitney U test.

**Table 2:** Baseline characteristics of patients in two cohorts.

Characteristics	Training cohort		Test cohort		*P*-value
	Metastasis (−) (n = 23)	Metastasis (+) (n = 53)	Metastasis (−) (n = 11)	Metastasis (+) (n = 22)	
Age (mean ± SD)	54.57 ± 9.60	56.3 ± 10.12	58.09 ± 12.24	55.55 ± 8.44	0.765^1^
FIGO stage (%)					0.273^2^
I–II	11 (47.8%)	18 (34.0%)	4 (36.4%)	5 (22.7%)	
III–IV	12 (52.2%)	35 (66.0%)	7 (63.6%)	17 (77.3%)	
Histological type (%)					0.596^2^
HGSC	16 (69.6%)	45 (84.9%)	8 (72.7%)	17 (77.3%)	
Non-HGSC	7 (30.4%)	8 (15.1%)	3 (27.3%)	5 (22.7%)	
Menopause (%)					0.371^2^
Yes	17 (73.9%)	39 (73.6%)	7 (63.6%)	15 (68.2%)	
No	6 (26.1%)	14 (26.4%)	4 (36.4%)	7 (31.8%)	
CA125 [median (IQR),U/ml]	535 (120.4–2035.0)	965.4 (241.6–2959.4)	323.7 (113.3–1158)	328.2 (119.7–2636.9)	0.265^3^
CA199 [median (IQR),U/ml]	10.0 (7.8–32.6)	14.7 (7.7–29.9)	9.3 (7.2–29)	25.2 (10.4–48.7)	0.421^3^
Location (%)					0.467^2^
Unilateral	14 (60.9%)	24 (45.3%)	6 (54.5%)	13 (59.1%)	
Bilateral	9 (39.1%)	29 (54.7%)	5 (45.5%)	9 (40.9%)	

*SD* standard deviation, *FIGO* International Federation of Gynecology and Obstetrics, *HGSC* high-grade serous carcinoma, *CA199* Carbohydrate antigen 199, *CA125* carcinoma antigen 125, *IQR* interquartile range.

^1^Student t test.

^2^Pearson’s chi-square test or Fisher test.

^3^Mann-Whitney U test.

Seventy-five patients (68.8%) were demonstrated to have metastasis by surgery and histology. The main metastatic sites included the uterus (n = 22), bladder (n = 15), appendix (n = 6), lymph nodes (n = 16), peritoneum (n = 52), greater omentum (n = 37) and intestinal canal (n = 58). A total of 206 metastases occurred in 75 patients with metastasis, with the peritoneum and intestinal canal being the two sites with the most metastases.

### Constructed radiomics model

**Figure 3:**
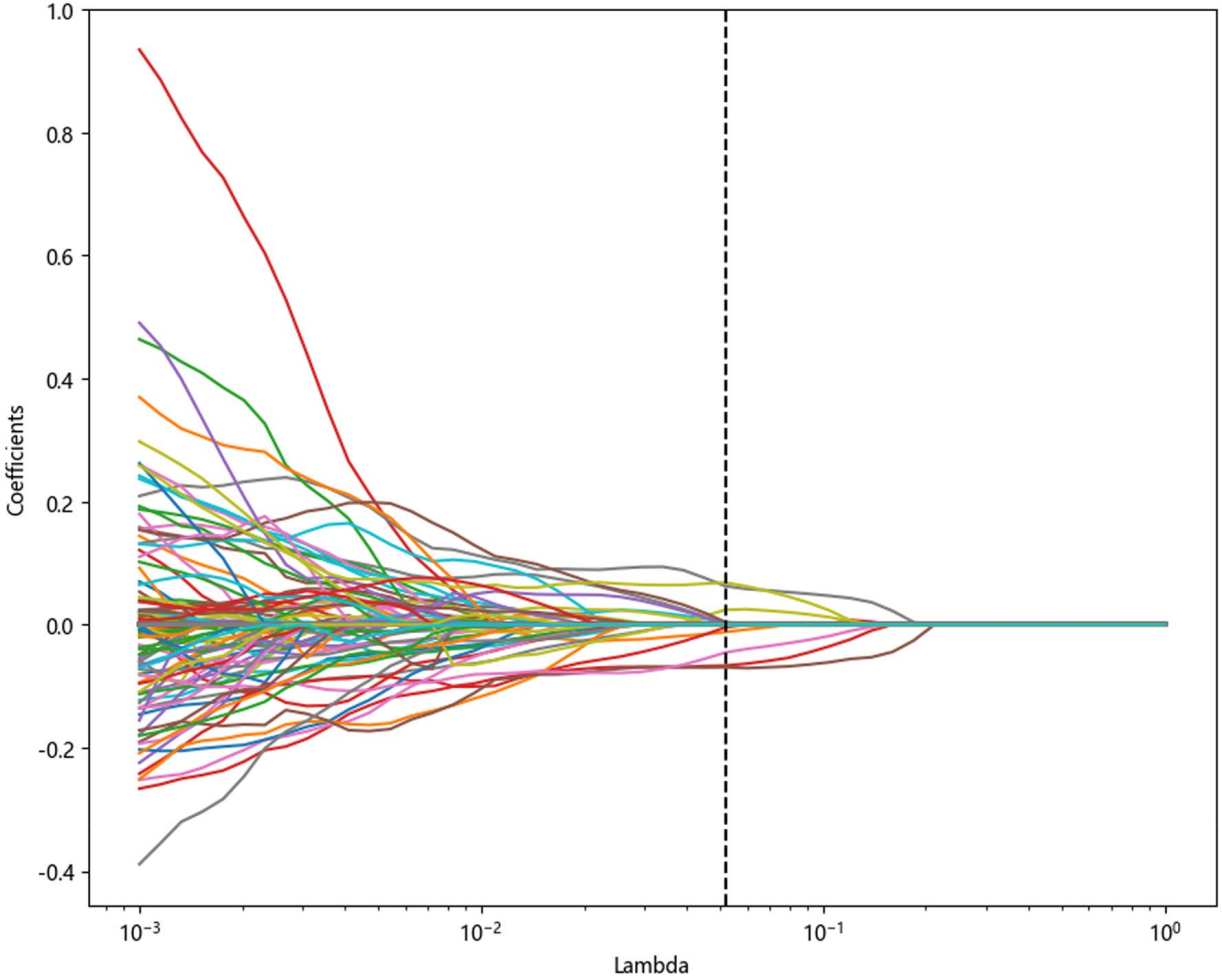
The process of the least absolute shrinkage and selection operation regression (LASSO) for feature selection. The color line represents the coefficient of the feature with λ Value change curve, corresponding to dashed line λ Value is the best λ Value, keep the 11 features where the coefficient is not 0.

LASSO method which was tunned using five-fold cross-validation on 76 patients in the training cohort analysis, was used for feature screening. The best λ Value (Fig. 3) was determined, and it was finally decided to retain 11 radiomics features (see Appendix Fig. A1). Using these features and their corresponding coefficients, the radscore was computed using the following formula:

$$ \begin{aligned} {\text{Rad score}} = & 0.6725226282143487 \\ & - 0.141426 \times {\text{Sphericity}} \\ & + 0.029083 \times {\text{LongRunLowGrayLevelEmphasis}} \\ & + 0.067196 \times {\text{MCC}}.3 \\ & + 0.001326 \times {\text{MaximumProbability}}.3 \\ & - 0.007038 \times {\text{SizeZoneNonUniformity}}.3 \\ & - 0.027125 \times {\text{DependenceNonUniformityNormalized}}.6 \\ & - 0.004618 \times {\text{RunVariance}}.6 + 0.000546 \times {\text{ZoneEntropy}}.6 \\ & - 0.006516 \times {\text{GrayLevelNonUniformity}}.23 \\ & - 0.058520 \times {\text{Busyness}}.7 \\ & + 0.063782 \times {\text{ MCC}}.8. \\ \end{aligned} $$

The detailed information of the radscore with EOC patient were shown in Appendix Fig. A2. The logistic regression classification algorithm was used to construct radiomics model. The radiomics model calculated AUCs of 0.920 (95% CI 0.845–0.991) in the training cohort, and 0.864 (95% CI 0.729–0.999) in the test cohort (Table 3).

**Table 3:** Performance evaluation of the models.

	Training cohort			Test cohort		
	AUC (95% CI)	Sen	Spe	AUC (95% CI)	Sen	Spe
Clinical model	0.843 (0.745–0.942)	0.943	0.652	0.860 (0.720–0.999)	0.864	0.818
Radiomics model	0.920 (0.845–0.991)	0.887	0.826	0.864 (0.729–0.999)	0.818	0.818
Combined model	0.929 (0.860–1)	0.943	0.826	0.909 (0.792–1)	0.864	0.909

*AUC* area under the curve, *95% CI* 95% confidence interval, *Sen* sensitivity, *Spe* specificity.

### Models comparison and clinical use

**Figure 4:**
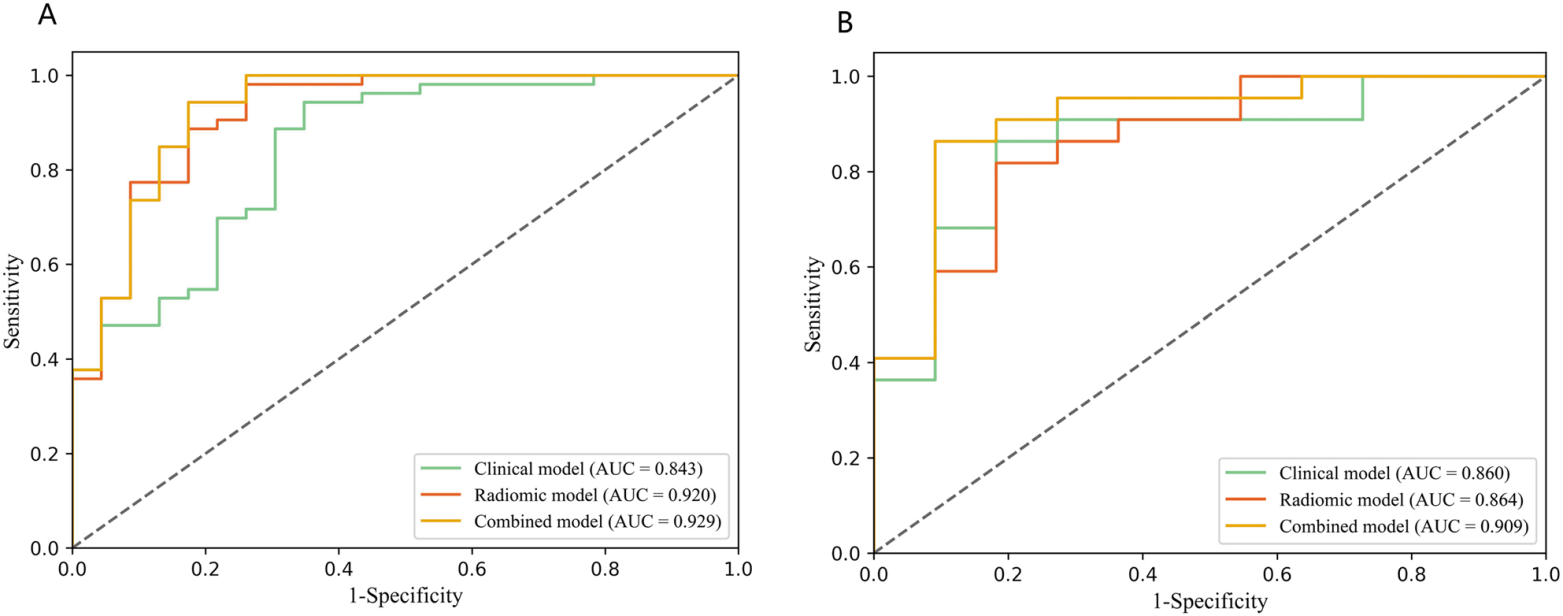
The receiver operating characteristic (ROC) curves of the radiomic model, the clinical model, and the combined model in training cohort (**A**) and test cohort (**B**). *AUC* area under the curve.

**Figure 5:**
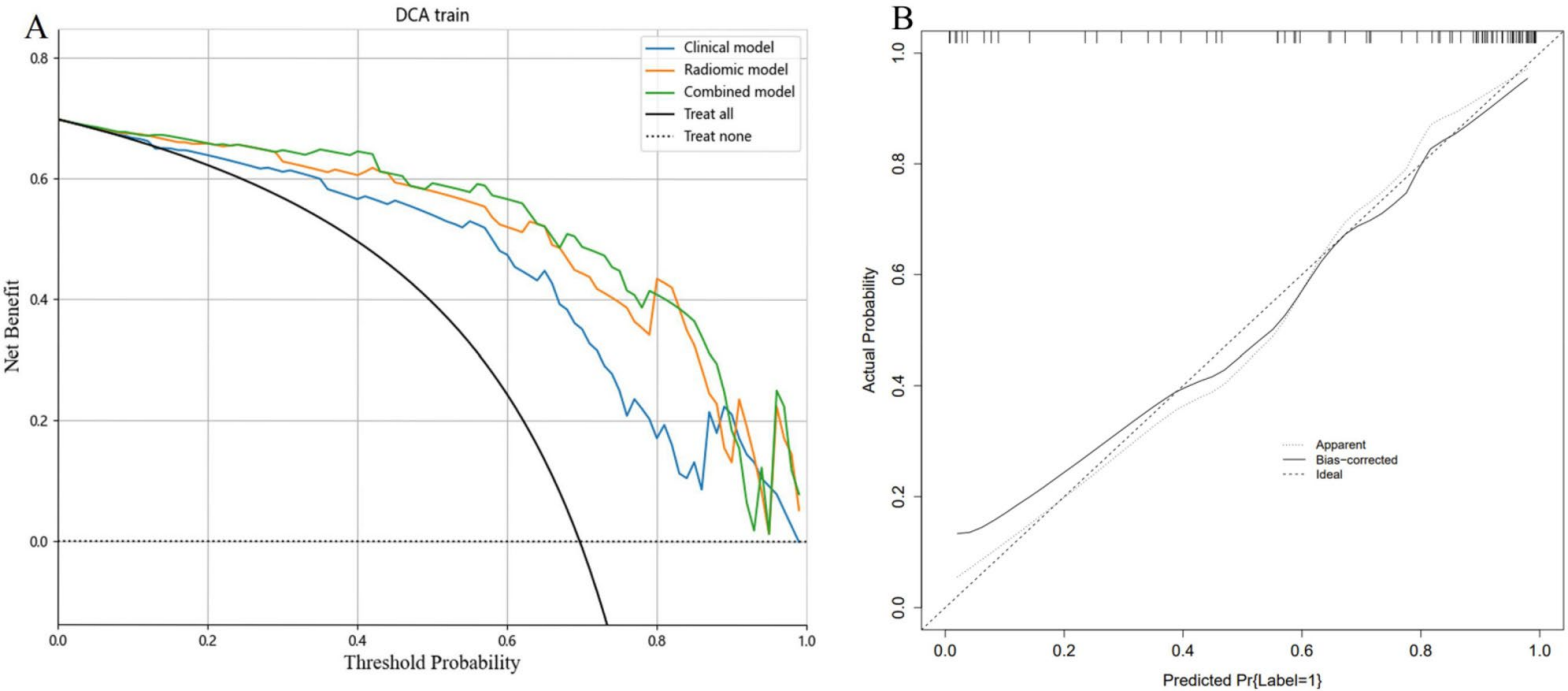
(**A**) Decision curves analysis of each model for predicting metastasis in the training cohort. The blue line represent the clinical model. The orange line represent the radiomics model. The green line represent the combined model. The black line represent the assumption that all patients have metastasis. The dotted line indicate the hypothesis that no patients have metastasis. (**B**) Calibration curves for the nomogram to predict metastasis in the training cohort. Diagonal dotted lines indicate perfect predictions, while dotted lines indicate a nomogram's performance. Closer fitting to the diagonal dotted lines indicates better performance. *DCA* decision curve analysis.

**Figure 6:**
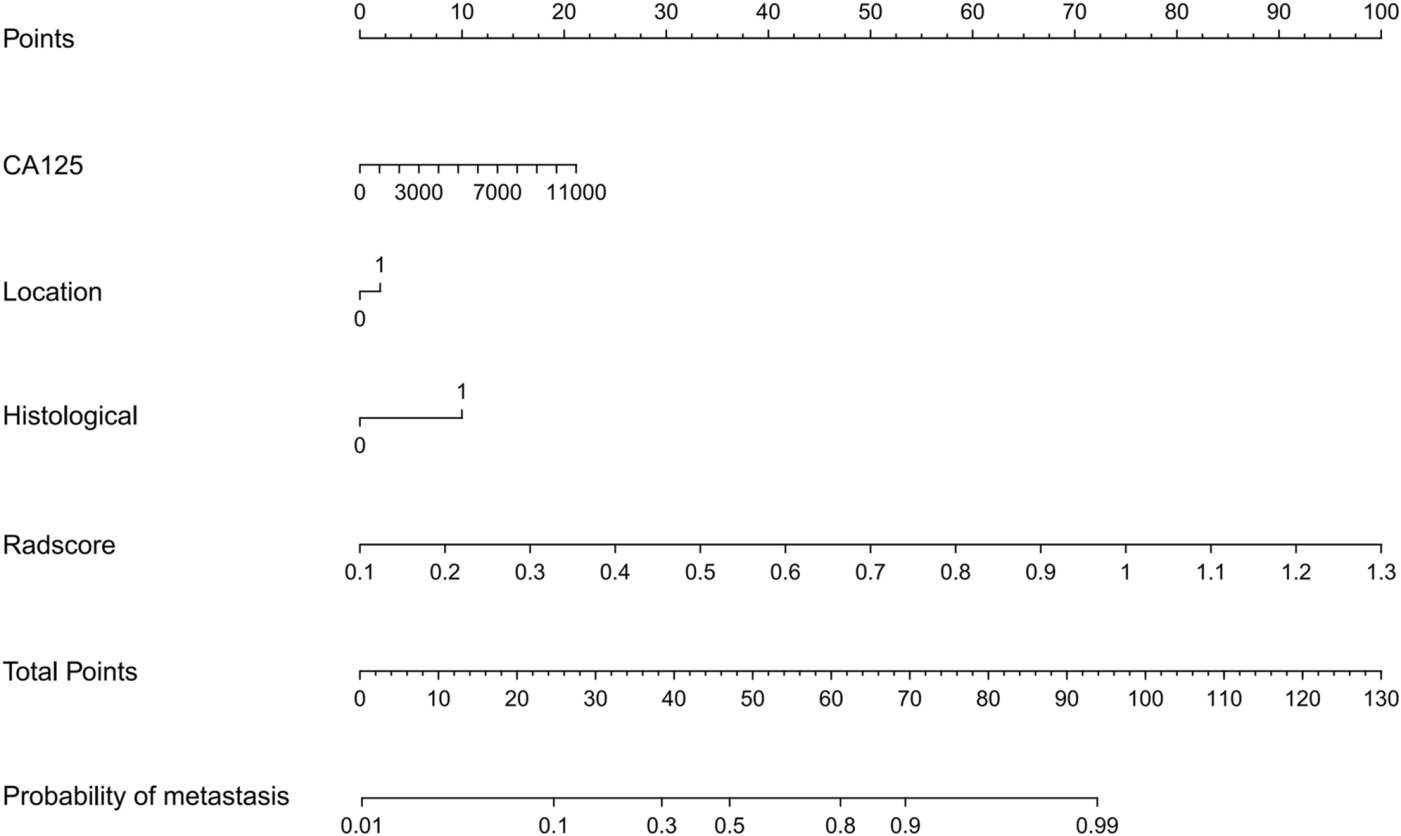
The radiomics nomogram. The radiomics nomogram, combining radscore, histological type, CA125, and tumor location, developed in the training cohort.

Three clinical characteristics with nonzero coefficients were obtained, including histological type, CA125, and tumor location. The logistic regression classification algorithm was used to construct three models. The clinical model calculated an AUC of 0.843 (95% CI 0.7447–0.9419) in the training cohort and 0.860 (95% CI 0.7196–0.9994) in the test cohort (Table 3). The ROC curves were used to assess the performance of the three models (Fig. 4). As shown in Table 3, the combined model outperformed the clinical model and radiomic model with a higher AUC of 0.929 (95% CI 0.860–1; accuracy: 89.5%; sensitivity: 94.3%; specificity: 82.6%) in training cohort and 0.909 (95% CI 0.792–1; accuracy: 87.9%; sensitivity: 86.4%; specificity: 90.9%) in test cohort. The DeLong test showed that the AUC value of the radiomics model was higher than that of the clinical model in the training cohort (*P* = 0.023). There was no significant difference between the radiomics model and the clinical model in the test cohort (*P* = 0.071). The DCA indicated that the combined model had the most significant net benefit when the threshold probability exceeded 15%, surpassing the benefits of both the treat-all and treat-none strategies (Fig. 5A). To facilitate clinical use, a radiomic nomogram was built by combing the histological type, CA125, tumor location with rad-score (Fig. 6). Calibration curves of the combined model showed a good consistency between the observed value and predicted value in the training and test cohorts (Fig. 5B).

## Discussion

In this present study, we constructed a radiomics nomogram including rad-score and clinical characteristics to evaluate the metastasis risk of EOC patients. The radiomics nomogram calculated AUCs of 0.909, which has better diagnostic performance than the clinical model (AUC, 0.860) and the radiomics model (AUC, 0.864). Therefore, the nomogram can be used to help clinicians formulate personalized treatment plans for EOC patients.

Out of the 109 patients enrolled, 75 had metastasis. This is consistent with the reported diagnosis of advanced disease in more than 70% of EOC patients. Metastasis appears to be a significant cause of incidence rate and mortality in patients with EOC^24^. Even though CA125 is an important prognostic factor for ovarian cancer stage and postoperative recurrence, disease progression, its sensitivity has been called into question^25^. Serum CA125 levels rise in approximately 80% of patients with advanced ovarian cancer^26,27^. Recent studies have analyzed that CA125 and pelvic fluid are independent risk factors for predicting peritoneal metastasis of ovarian cancer, with an AUC value of 0.686^5^. In our study, three clinical characteristics with nonzero coefficients were obtained, including histological type, CA125, and tumor location. The CA125 level is the feature that has the greatest diagnostic ability for evaluating EOC metastasis status. The higher the CA125 level, the greater the probability of diagnosing metastasis. The diagnostic probability of metastasis in high-grade serous carcinoma (HGSC) patients is higher than that in non-HGSC patients. The clinical model calculated an AUC of 0.843 (95% CI 0.7447–0.9419) in the training cohort and 0.860 (95% CI 0.7196–0.9994) in the test cohort.

Radiomics is a new non-invasive technology that employs medical image analysis and data mining techniques^28,29^. It is becoming an increasingly main component in accurate diagnosis and oncology, with the growth of advanced machine learning methods^30,31^. Radiomics based on MRI has been applied to predict preoperative peritoneal metastasis in ovarian cancer^5^. However, due to its contraindications, time commitment and higher costs, MRI is less beneficial for long-term follow-up and prognosis evaluation of ovarian cancer than CT. In contrast, early recurrence can be detected by CT-based radiomics features in patients with high-grade serous ovarian cancer^22^, and patients with residual tumors of ovarian cancer can be predicted to progress within 12 months^32^. Furthermore, enhanced CT scanning can better reflect the heterogeneity of tumor lesions. Since the degree of enhancement is an important indicator of the vascularization of the lesions, the radiomics based on enhanced CT scanning can obtain more information about tumor heterogeneity than that based on conventional scanning^33,34^. To our knowledge, few studies have used enhanced CT radiomics to evaluate the preoperative metastatic risk of EOC patients. Our findings demonstrate that radiomics-based machine learning classifier perform well in diagnosing EOC metastatic risk.

In this study, eleven radiomics features were finally selected using the LASSO method, which were ultimately employed to predict the metastasis of EOC patients. The majority of these features (10/11, 90.9%) comprised texture features (GLRLM, GLDM, GLCM, NGTDM and GLSZM) obtained from wavelet images. The remaining feature was a shape feature. Previous studies have indicated that these features, such as GLCM, GLRLM and GLDM can accurately assess intratumoral heterogeneity by quantifying both gray-level values and spatial complexity, and have diagnostic significance for metastasis^35,36^. Therefore, the radiomics features could noninvasively predict the metastasis status by capturing the differences in tumoral heterogeneity between EOCs with and without metastases^5^. In this research, the radiomics model achieved an AUC of 0.864 in the test cohort, which indicates that CT-based radiomics could serve as a more useful quantitative method for diagnosing metastasis.

Radiomics has been applied in the diagnosis of malignant tumors, evaluation of efficacy, and prediction of recurrence risk. Chen et al.^37^ indicates that CT-based radiomics can predict the risk of lymph node metastasis in EOC patients, especially for some occult lymph node metastases. Accurately predicting the FIGO staging of EOC before treatment is crucial in formulating effective treatment strategies. Our study constructed a radiomics model by extracting high-throughput information from the enhanced CT images. The radiomics model calculated AUCs of 0.920 (95% CI 0.845–0.991) in the training cohort, and 0.864 (95% CI 0.729–0.999) in the test cohort. Compared to previous studies, our radiomics model has demonstrated higher diagnostic efficiency^38^. In order to make the results more intuitive, we established a radiomics nomogram that combines clinical features and rad score. The radiomics nomogram has shown a strong predictive performance with AUCs of 0.929 in the training cohort and 0.909 in the test cohort. Our findings suggest that the radiomics nomogram can be used as a reliable and accurate method to predict metastasis in EOC patients, thereby assisting clinicians in improving their diagnostic abilities. In the training cohort, when the threshold is set within the range of 15–80%, the decision curve of the radiomics nomogram is farthest from the Treat None and Treat All lines. Therefore, radiomics nomogram has the strongest clinical practicality and can help determine who should receive treatment by calculating net benefits.

Machine learning is a subset of artificial intelligence, with three main types: supervised learning, unsupervised learning, and reinforcement learning. Deep learning is a form of machine learning, mainly applied in areas such as image segmentation and detection, image classification, clinical outcome prediction^39,40^. Convolutional neural network (CNN) is the most widely used deep learning technique, with a more powerful architecture and flexible configuration, which can learn more discriminative features to achieve more accurate detection^41^. CNN can automatically extract useful features from medical images, which are crucial for diagnosis and treatment decisions, and its application in the field of oncology is becoming increasingly widespread.

Although the study has achieved significant results, it also has several limitations. Firstly, it is a single center study with a relatively small sample size. Although the research results demonstrate the superior prediction performance of the radiomics nomogram, it is necessary to collect larger samples to comprehensively evaluate the generalization ability of radiomics models in the future. Secondly, conducting radiomics analysis solely based on CT arterial phase images may affect the accuracy of the results. In the future, other phase images will be considered for comparative analysis. Finally, it is not possible to completely eliminate the interference of volume effect since the tumor boundary is manually delineated. One of the future research directions will involve exploring automatic segmentation techniques for tumor lesions.

## Conclusion

In summary, we developed a radiomics nomogram that combined the rad score and clinical characteristics, which helps evaluate metastasis in EOC patients. Compared with clinical model and radiomics model, the nomogram demonstrates significantly superior diagnostic performance, which can serve as a useful and convenient tool to assist clinicians in developing personalized treatment plans for EOC patients.

### Supplementary Information

Supplementary Figures.

Supplementary Information.

## Data Availability

The original contributions presented in the study are included in the article/Supplementary Material. Further inquiries can be directed to the corresponding authors.

## Abbreviations

EOC: Epithelial ovarian cancer
PET: Positron emission tomography
MRI: Magnetic resonance imaging
CT: Computed tomography
PACS: Picture archiving and communication system
CA125: Carcinoma antigen 125
CA199: Carbohydrate antigen 199
FIGO: International Federation of Gynecology and Obstetrics
VOI: Volume of interest
GLCM: Gray-level co-occurrence matrix
GLRLM: Gray-level run length matrix
GLSZM: Gray-level size zone matrix
GLDM: Gray-level dependence matrix
NGTDM: Neighborhood gray-tone difference matrix
ICC: Intraclass correlation coefficient
mRMR: Minimum redundancy maximum relevance
LASSO: Least absolute shrinkage and selection opera-tion regression
Rad-score: Radiomics score
AUC: Area under curve
ROC: Receiver operating characteristic curve
DCA: Decision curve analysis
HGSC: High-grade serous carcinoma
CNN: Convolutional neural network
