# Supplementary material for: Development and validation of radiomics nomogram for metastatic status of epithelial ovarian cancer
Source: Sci Rep. 2024 May 30;14:12456. doi: 10.1038/s41598-024-63369-1 (PMC11139946; doi:10.1038/s41598-024-63369-1)
Supplement: Supplementary file 1 — Supplementary Figures. [file 41598_2024_63369_MOESM1_ESM.docx]

Appendix Figures

Appendix Figure A1


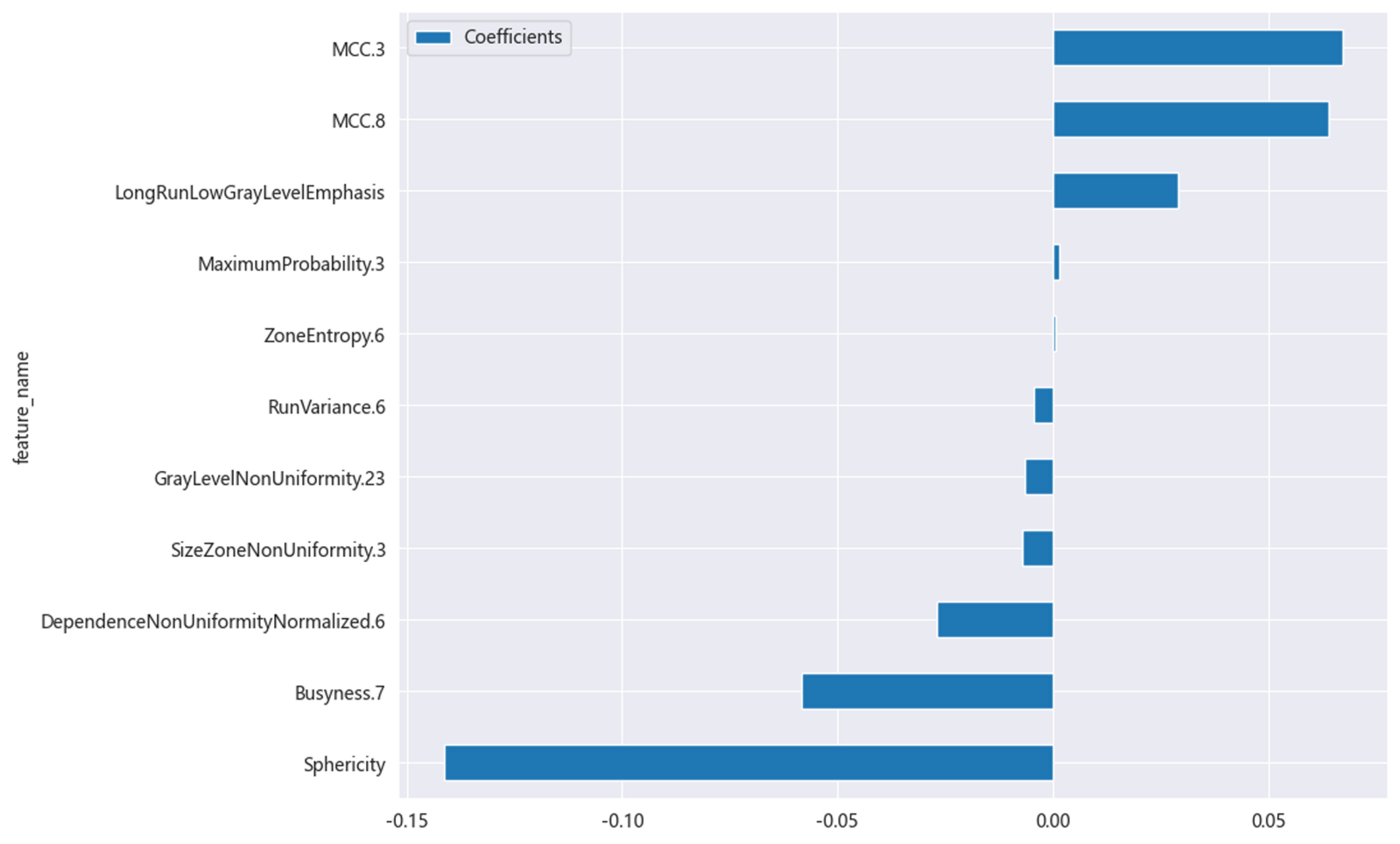


Appendix Figure A1. The eleven radiomic features with nonzero coefficients are indicated in the plot. The x-axis represents its coefficient in lasso regression. The larger the coefficient, the higher the prediction value.

Appendix Figure A2


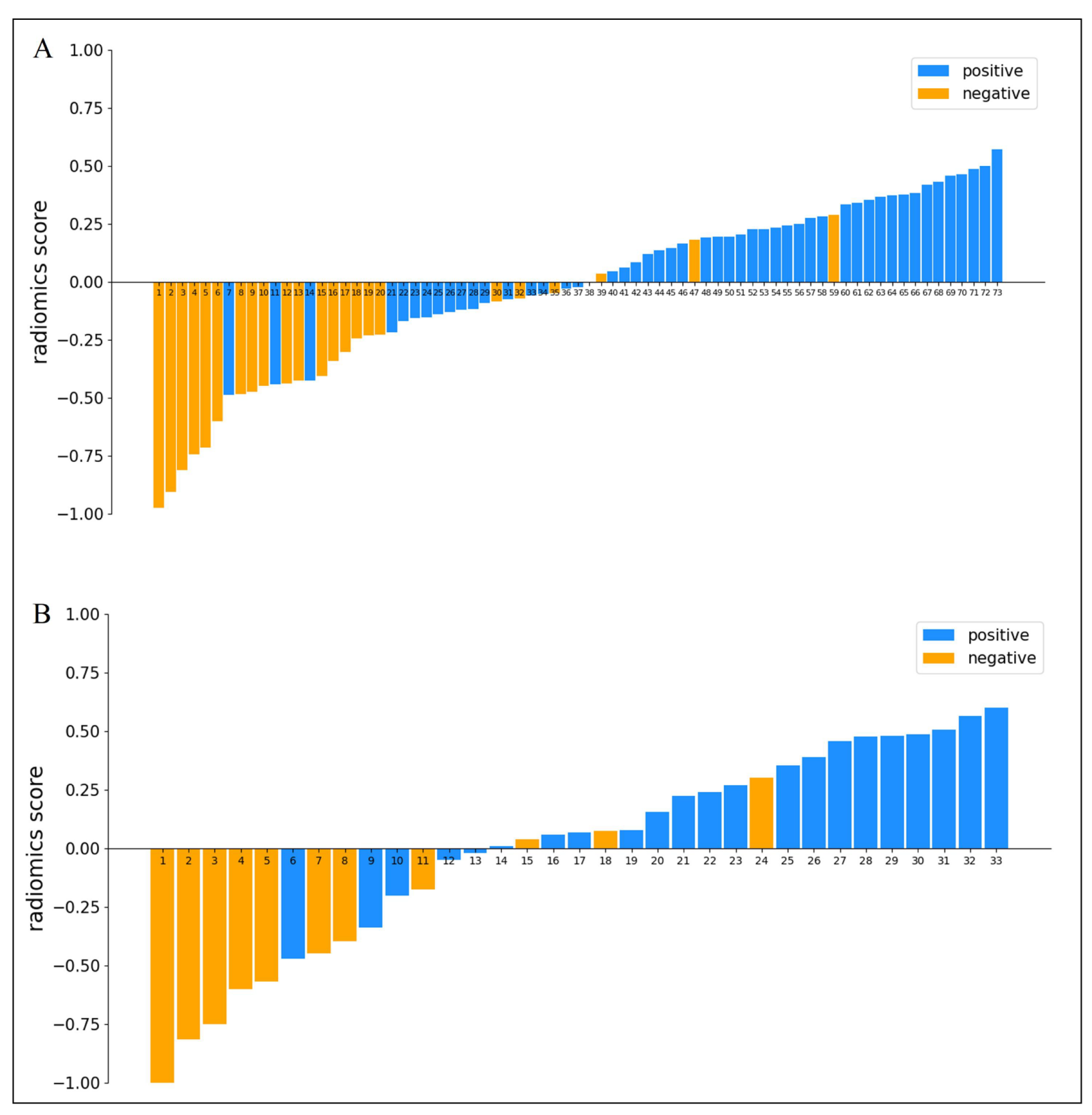


Appendix Figure A2. Radiomic score of radiomic feature for each patient in the training cohort (A) and test cohort (B).
