# Supplementary material for: Development and validation of radiomics nomogram for metastatic status of epithelial ovarian cancer
Source: Sci Rep. 2024 May 30;14:12456. doi: 10.1038/s41598-024-63369-1 (PMC11139946; doi:10.1038/s41598-024-63369-1)
Supplement: Supplementary file 2 — Supplementary Information. [file 41598_2024_63369_MOESM2_ESM.docx]

**Appendix A1**

Radiomics features selection was built with the Python Scikit-learn package (Python version 3.7, Scikit-learn version 1.1.2, <http://scikit-learn.org/>). Statistical tests were performed using R statistical software version 3.5.2. The ROC curves were plotted using the "pROC" package. We used the "RMS" package to perform Nomogram construction and calibration plots. The Hosmer–Lemeshow test was conducted using the "generalhoslem" package. DCA was performed using the " rmda " package.
